# Pre-Participation Physical Fitness does not Influence Adherence to a Supervised Exercise Program

**DOI:** 10.5935/abc.20170132

**Published:** 2017-10

**Authors:** Fábio Akio Nishijuka, Christina Grüne de Souza e Silva, Carlos Vieira Duarte, Claudio Gil Soares de Araújo

**Affiliations:** 1 Programa de Pós-Graduação em Cardiologia - Universidade Federal do Rio de Janeiro (UFRJ), Rio de Janeiro, RJ - Brazil; 2 Clínica de Medicina do Exercício (CLINIMEX), Rio de Janeiro, RJ - Brazil

**Keywords:** Muscle Strength, Oxygen Consumption, Physical and Rehabilitation Medicine, Sports Medicine

## Abstract

**Background:**

Exercise-based cardiac rehabilitation tends to reduce mortality. However, it requires medium/long-term adherence to regular physical exercise. It is relevant to identify the variables that affect adherence to an supervised exercise program (SEP).

**Objective:**

To evaluate the influence of pre-participation levels of aerobic and non-aerobic physical fitness components in medium-term adherence to SEP.

**Methods:**

A total of 567 SEP participants (65 ± 12 years) (68% men) were studied. Participants adherent to the program for less than 6 months (48%) (non-adherent - NAD) were compared with 52% of participants who were adherent for 6 months or more (adherents - AD). In the non-aerobic fitness, flexibility (FLX) (Flexitest) and muscle power (MPW)/body weight in standing rowing (watts/kg) were evaluated while aerobic fitness was obtained by direct measure of VO_2_max/body weight (VO_2_). These measurements were normatized for sex and age based on percentiles (P) (P-FLX/P-MPW) of reference data or percentages of predicted (P-VO_2_). Additionally, AD and NAD with extreme results (tertiles) were simultaneously compared for the three variables.

**Results:**

There was no difference between AD and NAD for non-aerobic results, in median [P25-P75], P-FLX: 30 [13-56] and 31 [9-52], respectively, (p = 0.69) and P-MPW: 34 [17-58] and 36 [16-62], respectively (p = 0.96), and for aerobic results (mean ± standard error) P-VO_2_ (75.9 ± 1.3% and 75.0 ± 1.3%, respectively) (p = 0.83). When comparing extreme tertiles, a difference was found for P-MPW in the lower tertile only, with a slight advantage of AD over NAD- 9 [5-16] versus 4 [1-11] (p = 0.04).

**Conclusion:**

Although awareness of the pre-participation levels of aerobic and non-aerobic physical fitness components is useful for individualized exercise prescription, these variables do not seem to influence medium-term adherence to SEP.

## Introduction

The beneficial effects of regular physical activities and physical exercises, exercises on health, even if in small doses, are widely known.^1^ High levels of aerobic^2^ and anaerobic^3^ fitness are associated with reduced all-cause mortality in middle-aged and elderly individuals. In contrast, there is evidence that only three weeks of bed rest would result in a reduction of aerobic fitness by 30%.^4^

In fact, attention has been increasingly focused on physical exercise for secondary prevention of cardiovascular diseases (CVD) since the end of the 50s.^5-7^ Currently, physical exercise is recommended by the guidelines of cardiology societies all over the world^8-12^ as part of the so called cardiac rehabilitation (CR). CR encompasses several components, but classically, physical exercise in different forms are the single or the main component of which is characterized as exercise-based CR.^13,14^ Although underused and frequently of short duration, exercise-based CR promotes several benefits to health, especially in the reduction of cardiovascular mortality.^13^ However, despite these favorable evidence of the exercise-based CR, staying physically active throughout life, i.e., being adherent to habitual exercise is difficult for the majority of CVD patients,^15,16^ reducing the potencial benefits of this intervention.

Therefore, it seems relevant to investigate the variables capable of influencing the adherence rate to adherence to an supervised exercise program (SEP).^17-21^ To the best of our knowledge, the possible influence of the aerobic and non-aerobic^22^ fitness component levels before SEP on its adherence has not been studied yet. If, on the one hand, it may be easier to increase initially low fitness levels, on the other hand, individuals with low physical fitness may feel unable to exercise regularly, which could compromise their adherence to a SEP. In this context, we aimed to investigate the influence of flexibility (FLX), muscle power (MPW) and aerobic fitness or maximum oxygen uptake (VO_2_max) on medium-term adherence to a SPE.

## Methods

### Sample

We retrospectively analyzed data of 644 individuals who initiated their participation in a SEP in a private clinic located in the south of Rio de Janeiro city, Brazil, between January 2009 and March 2015. These individuals had been mostly referred by their assistant physicians. Before initiating the SEP, participants had undergone a comprehensive and detailed assessment, including anamnesis, physical exercise, anthropometry, electrocardiography, resting spirometry, 4-second exercise test, cardiopulmonary exercise test (CPET),^23,24^ and evaluation of FLX^25^ and MPW.^26^

For final characterization of the sample, individuals with one of the following conditions were excluded: 1- age younger than 30 years; 2- an interval longer than 120 days between pre-participation assessment and the start of participation in the SEP; 3- absent data on FLX, MPW or VO_2_max in the pre-participation assessment. In the pre-participation assessment. After application of these criteria, 6 volunteers were excluded because of age, 14 for having started the SEP 120 days after pre-participation assessment, 41 for having incomplete non-aerobic fitness data, and 16 for not performing CPET or for having not achieved maximum strength. Therefore, 567 participants were included in the study.

For analysis if adherence, an 'appropriated participation was considerated as a continuos participation for more than six-months, that is, a medium-term participation without interruptions longer than one-month. There was a wide variation in the frequency of attendance of the SEP - one to six sessions a week - even though most were advised to attend the SEP three days a week. Thus, different from some other studies, the number of sessions that the volunteers effectively participated was not considered to characterize adherence. Participants were then separated in two groups according to the period of continuous participation in this SEP, as determined by registration in the attendance forms non-adherents (NAD) - less than six months - adherents (AD) - six months or more - regardless of the number of sessions attended in each month during the study period (January 2009 - September 2015).

All participants read and signed the informed consent form before the CPET and participation in the SEP. Both informed consent form and the retrospective analysis of the data for research purposes were approved by the Ethics Committee (report number 218/10).

### Supervised exercise program

The SEP was conducted in a temperature- (21-24°C) and humidity-controlled (4-60%) room. Before initiating the exercise session, each participant was briefly seen by a physician, who prescribed the aerobic part of the session. The sessions included aerobic exercises - cycle ergometer tests of lower and upper limbs, treadmill, rowing ergometry, and ski ergometer test - and exercises for muscle strengthening, FLX, balance and motor coordination, each session with 60 to 75 minutes of duration. According to the patients clinical conditions and individual goals, some participants also underwent inspiratory muscle training and isometric handgrip test, whose clinical safety has been previously demonstrated.^27,28^ Continuous heart rate monitoring and intermittent blood pressure and electrocardiohgram monitoring were performed during the exercise sessions, as clinically indicated.

One important characteristic of this SEP, and a variable that could positively contribute to adherence, was the complete freedom of choosing for exercising anytime - 15.5 hours/day during the weekdays and 9 hours/day on Saturdays - during the operation time of the clinic, a total of 86.5 hours per week.

### Assessment of physical fitness components: FLX, MPW and aerobic conditioning

FLX was assessed by Flexiteste,^29,30^ which evaluates the maximum passive mobility of twenty joint movements, including seven joints, using an increasing, ordinal scale of scores ranging from 0 to 4, by comparing the amplitude obtained by each patient with the specific evaluation maps. The sum of the scores of each of the 20 joint movements generated a global index of body flexibility named Flexindex. Aiming to control the influence of age and sex, Flexindex of each participant was expressed in percentile (P) (P-FLX), adjusted for age and sex, based on data from previous report.^25^

Assessment of relative MPW - MPW (watts)/body weight (kg) - was performed during the concentric phase in standing position, using a standardized method described in details in previous studies, showing the reliability of the evaluations.^26^ Briefly, MPW was measured using the Fitrodyne (Fitronic, Slovakia), by the product of mean velocity (m/s) during concentric exercise and weight lifted (kg). The weight was gradually increased every five kilograms until maximum MPW was achieved.^26,31^ Similarly to FLX, individual data were adjusted using laboratory reference data (unpublished data obtained from 4,567 adults in both sexes and age range compatible with the present study), and expressed as percentile (P-MPW), according to age and sex.

Aerobic fitness was evaluated by CPET using directly measured VO_2_max relative to body weight and direct analysis of expired gases (VO2000; Medgraphics, USA), as previously described in details^24,32^ and following a recent guidelines of Brazilian authors.^23^ All tests were performed by only four physicians in a temperature-controlled room, which was properly equipped for potential clinical events. Tests were performed using individualized ramp protocols, aiming a duration of 8 to 12 minutes to achieve exhaustion.^33^ Individual aerobic fitness, in mL/(kg.min), was then expressed as percentage of predicted VO_2_max (P-VO_2_), which was calculated by the formula 60-0.55 x age (years) for men and 48-0,37 x age (years) for women.^34^

### Statistical analysis

Statistical analysis was performed based on the measuring scales and data distribution. The D'Agostinho & Pearson, Shapiro-Wilk and Kolmogorov-Smirnov tests were used to test the normality of data distribution. The Student's t-test was used for comparisons of continuous, normally distributed variables between groups and between subgroups. The Mann-Whitney test was used for analysis of continuous variables without normal distribution, and the chi-square statistics for categorical variables (clinical features and use of medications). Results are shown as mean and standard error for continuous, normally distributed variables, and as median and interquartile range (25th-75th percentile) or percentage (as appropriate) for the others.

As an additional analysis, we applied the Mann-Whitney test to identify, in NAD and AD groups, possible differences in adherence to SEP in those participants located in the lower (first tertile) and upper (third tertile) limits of physical fitness range of the three physical fitness components. Thus, new, smaller subgroups were defined - NAD1 and AD1, for NAD and AD individuals, respectively, located in the first tertile; and NAD3 and AD3, for non-adherent and adherent individuals, respectively, located in the third tertile - with results already adjusted for age and sex in flexibility, muscle power, and aerobic fitness. The GraphPad Prism 6.0 (GraphPad Software, USA) was used for analyses and figures, and a level of 5% was set as statistically significant.

## Results

Among the 567 participants (68% men), mean age was 65 ± 12 years (31-92 years). Based on the criterion used to define adherence to SEP, i.e. continuous attendance in the program for six months, 52% were classified as AD and 48% as NAD. There were no differences in age (p = 0.29) or sex distribution (p = 0.27) between AD and NAD. Body mass index (BMI) varied from 17.5 to 52.4 kg/m^2^ (median 27.1 kg/m^2^, interquartile range of 24.6 - 30.5 kg/m^2^), without difference between the groups (p = 0.25).

Based on clinical data obtained from patients' medical records, 61% of patients were hypertensive, 56% had coronary artery disease (CAD), 31% had previous acute myocardial infarction, 37% had a history of percutaneous angioplasty and 17% of coronary artery bypass surgery. In addition, 21% were obese, 30% had a diagnosis of diabetes mellitus, 46% were sedentary, 55% were former smokers, i.e., had not smoked for at least six months, and only 5% were active smokers. Considering all these variables, there was only a, slight difference in smoking history (mostly former smokers) between AD (55%) and NAD (65.8%) (p = 0.01). With respect to current and regular use of medications, 63% of patients used beta-blockers, 76% used hypolipidemic agents, 73% used antiplatelet gents, and 59% used psychotropics, with no difference between the groups (p > 0.05). A more detailed description of these results is found in Table 1.

**Table 1:** Clinical characteristics and use of medications in adherent and non-adherent patients (n = 567) to the supervised exercise program (SEP) and in the subgroups in the lower (n = 43) and upper (n = 50) extreme tertiles of aerobic and non-aerobic physical fitness

	Participants			1^st^ (lower) tertile			3^rd^ (upper) tertile		
	AD (n = 298)	NAD (n = 269)	p	AD1 (n = 18)	NAD1 (n = 25)	p	AD3 (n = 20)	NAD3 (n = 30)	p
**Clinical features**									
Coronary artery disease (%)	58	53	0.24	22	48	0.08	50	57	0.64
Systemic arterial hypertension (%)	64	58	0.16	67	80	0.32	65	57	0.56
Dyslipidemia (%)	69	68	0.78	50	76	0.08	80	70	0.43
Diabetes mellitus (%)	30	29	0.90	44	60	0.31	10	23	0.23
Smoking history (%)	55	66	0.01	44	64	0.20	50	70	0.15
Sedentary lifestyle (%)	44	48	0.30	72	72	0.99	25	33	0.53
**Use of medications**									
Beta-blockers (%)	66	60	0.17	72	72	0.99	65	63	0.90
Statins (%)	77	74	0.43	67	76	0.50	80	83	0.76
Antiplatelet agents (%)	73	72	0.64	50	68	0.23	60	77	0.21
Psychotropics (%)	58	60	0.79	56	64	0.58	30	50	0.16

NAD: non-adherents (<6 months of SEP); AD: adherents (≥6 months of SEP); NAD1: non adherent 1^st^ tertile; AD1: adherent 1^st^ tertile; NAD3: non-adherent 3^rd^ tertile; AD3: adherent 3^rd^ tertile. Comparison of data distribution of the variables was carried out by chi-square test.

The interval between pre-participation assessment and the first SEP session was between 1 and 9 days (median 4 days) for half of participants. During the study period, median duration of participation in the SEP was 6 months, with P25 and P75 of 3 months and 15 months, respectively. Median number of the SEP sessions attended by participants was 46, with P25 and P75 of 19 and 122 sessions, respectively, and minimum of one and maximum of 1,358 sessions. Most participants attended between 5 and 10 sessions per month, with a median of 7.6 sessions/month. Comparison of demographic and SEP's participation data between AD and NAD are shown in Table 2.

**Table 2:** Results of demographic data and attendance data of adherent and non-adherent participants (n = 567) to the supervised exercise program (SEP) and in the subgroups in the lower (n = 43) and upper (n = 50) extreme tertiles

	Participants			1^st^ (lower) tertile			3^rd^ (upper) tertile		
	AD (n = 298)	NAD (n = 269)	p	AD1 (n = 18)	NAD1 (n = 25)	p	AD3 (n = 20)	NAD3 (n = 30)	p
Men (%)	66	70	0.26	64	56	0.57	83	90	0.51
Age (†)	66 ± 0.7	64 ± 0.7	0.29	60 ± 2.8	57 ± 2.1	0.34	69 ± 1.8	70 ± 2.4	0.76
Body mass index (kg/m^2^) (†)	27 ± 0.2	28 ± 0.3	0.25	32 ± 1.5	34 ± 1.5	0.43	26 ± 0.74	25 ± 0.43	0.75
Interval between assessment and enrollment (days) (†)	9 ± 0.9	11 ± 1.2	0.44	7 ± 2.1	12 ± 4.9	0.73	14 ± 4.7	12 ± 4.6	0.28
Months of SEP (†)	22 ± 1.1	2.9 ± 0.1		19 ± 3.5	3 ± 0.3		22 ± 5.4	3 ± 0.2	
Number of sessions/month (*)	9 (7–10)	7 (4–9)	< 0.001	9 (9–13)	7 (5–9)	0.010	9 (7–10)	5 (3–8)	0.003

(*) median (percentile 25 – percentile 75);

(†) mean ± standard error, t-test for age and Mann-Whitney test for the other variables. Comparison between men and women percentiles was performed by the chi-square test.

NAD: non-adherents (< 6 months of SEP); AD: adherents (≥ 6 months of SEP); NAD1: non adherent 1^st^ tertile; AD1: adherent 1^st^ tertile; NAD3: non-adherent 3^rd^ tertile; AD3: adherent 3^rd^ tertile.

**Figure 1:**
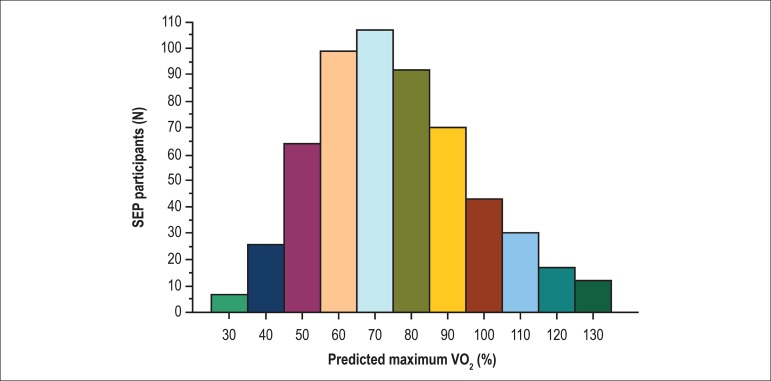
Distribution of aerobic fitness results (n = 567). SEP: supervised exercise program.

Regarding the results of physical fitness components in the pre-participation assessment, which are the main object of this study, we found that the values obtained in percentile and/or percentage of predicted value (adjusted for age and sex) for the 567 participants tended to be lower than those expected for the general population, i.e., percentiles equal to or greater than 50 (median) and percentage equal to or greater than 100%. For non-aerobic components the median and interquartile range were: P-FLX = 30[11-55] and P-MPW = 35[17-60], and for the aerobic component the mean ± standard error was P-VO_2_ = 75.5 ± 0.91. Distribution of aerobic results, expressed as P (%) of VO_2_max predicted, obtained by the CPET is shown in Figure 1. Comparison of the AD group with the NAD group showed no significant differences in the three components of aerobic and non-aerobic fitness, as described in Table 3.

**Table 3:** Results of aerobic and non-aerobic physical fitness in adherent and non-adherent participants (n = 567) and in subgroups in the lower (n = 43) and upper (n = 50) tertiles

	Participants			1^st^ (lower) tertile			3^rd^ (upper) tertile		
	AD (n = 298)	NAD (n = 269)	p	AD1 (n = 18)	NAD1 (n = 25)	p	NAD3 (n = 20)	NAD3 (n = 30)	p
Flexindex*	30 (13–56)	31 (9–52)	0.69	6 (1–11)	4 (1–11)	0.70	70 (60–88)	74 (49–92)	0.85
Relative power *	34 (17–58)	36 (16–62)	0.96	9 (5–16)	4 (1–12)	0.039	78 (64–92)	73 (64–87)	0.42
Predicted relative maximum VO_2_ (%)(†)	75.9 ± 1.27	75.0 ± 1.30	0.83	51.7 ± 2.65	52.1 ± 1.94	0.81	104.3 ± 3.54	103.0 ± 2.42	0.86

(*) age- and sex-percentile in median (25th percentile - 75^th^ percentil);

(†) percentile of predicted relative maximum VO_2_ in mean ± standard error, Mann‑Whitney test.

NAD: non-adherents (< 6 months of SEP); AD: adherents (≥ 6 months of SEP); NAD1: non adherent 1^st^ tertile; AD1: adherent 1^st^ tertile; NAD3: non-adherent 3^rd^ tertile; AD3: adherent 3^rd^ tertile; maximum VO_2_: maximum oxygen consumption

In the other analysis, patients with worse (lower tertiles, n = 48) and better (upper tertiles, n = 50) physical fitness (the three components together) were compared for adherence to the SEP. In analysis of clinical data, current and regular use of medications, and results of physical fitness components, the only significant difference was found in P-MPW for individuals located in the lower tertile (in median and interquartile range): AD1 = 9 (5-16) and NAD1 = 4 (1-12) (p = 0.04). These results are described in Tables 1 and 2.

## Discussion

The literature indicates that regular physical exercise is important for secondary prevention of CVD.^8,9^ However, a very small proportion of patients is referred to, and an even smaller proportion of patients are actually enrolled in formal programs of CR or SEP. Despite cost-effectiveness of these programs,^21^ the number of centers available in Brazil is known to be lower than the desired one. Among the individuals enrolled in the programs, a variable and unfortunately small number complete a reasonable number of exercise sessions, and an even smaller proportion effectively adopts the regular physical exercise as a healthy lifestyle practice. The present article contributes to the body of knowledge in the area, showing that pre-participation levels of the three components of both aerobic and non-aerobic physical fitness have no significant effect on medium-term (i.e., six months) adherence to a SEP.

Measurement and promotion of adherence to physical exercise is a big challenge that has been investigated for some decades, but there is still insufficient evidence towards desired clinical results.^35,36^ Probably, adherence to a SEP is influenced by many factors including cognitive, behavioral, and environmental factors. By adopting different approaches and temporal criteria for characterization of adherence and non-adherence, we showed in previous studies with participants of this same SEP, the negative effect of obesity on adherence,^19^ and that the distance from participants' home to the training center did not seem to be a determinant factor for adherence.^17^

Effective participation in SEPs normally results in significant improvement of physical fitness. A recent meta-analysis^37^ indicates that mean gain in aerobic fitness was 6.6 mL/(kg.min), and 43 of the 48 original studies included showed significant aerobic gains from participation in an exercise-based CR. In this line of thought, it is of note that initial aerobic physical fitness seems to have a prognostic influence among CR participants. For example, in a study on 12,169 men with CVDs, Kavanagh et al.^38^ observed that direct measurement of aerobic fitness before the CR had a strong, favorable influence on cardiovascular and all-cause mortality. Ross et al.^39^ demonstrated in a recently published review that aerobic fitness is closely related to morbidity and mortality, and is a stronger predictor of cardiovascular risk than traditional risck factors such as diabetes mellitus, arterial hypertension and smoking.

Nevertheless, despite the rich literature about adherence to exercise, there seem to be very few data related to a possible influence of pre-participation aerobic and non-aerobic physical fitness levels on adherence to a SEP or to a more comprehensive CR program. Besides, the pre-participation levels of aerobic physical fitness were the object of studies about other relevant clinical outcomes. In light of this, it is worth mentioning the meta-analysis performed by Sandercook et al.,^37^ which identified that initial aerobic levels may not predict the magnitude of absolute gain in VO_2_max when participating on a CR,^37^ although this seems to vary with the type of cardiovascular intervention and patient's clinical condition.^40^ Unfortunately, this meta-analysis did not include adherence and hence its results cannot be compared with ours.

In fact, one should recognize the existence of many clinical, logistic and methodological difficulties for a careful, broad assessment of physical fitness components of all candidates for the CR programs and SEPs. Therefore, to our knowledge, this is the first study to obtain direct measurements of aerobic fitness and FLX and MPW data from a large group of participants before starting the SEP, and to evaluate the influence of these results on medium-term adherence (six months) to a SEP. 

In the search of variables able to measure, in a practical, objective way, the chance of adherence, and to help physicians in the individualized approach of participants in the beginning of a SEP, we analyzed the possible influence of pre-participation levels of aerobic and non-aerobic components of physical fitness (FLX, MPW and aerobic fitness) on adherence to a SEP in a six-month period. Participants of the AD group and NAD group were not different in terms of sex, age, BMI, clinical profile and regular use of medications; the only exception was the different percentage of participants with history of smoking, which was higher in the NAD group. It is worth to mention that we opted to describe the variable 'smoking history' in the same way it has been usually described in clinical studies. However, in the present study, the percentage of active smokers among the SEP participants was very small, lower than 5%, with no relevant difference between the groups and subgroups of the study. 'Active smoking' could have influenced the results if there was a clinically relevant difference in this variable between the groups. With respect to age, our data differ from a recent study that demonstrated that adherence in elderly patients is lower than in younger patients.^41^ Demographic and clinical features, as well as different SEP may explain the discrepancy in these results.

In the general population, predicted VO_2_max is expected to be 100%, and the mean percentile for age and sex is expected to be 50 (p50). However, our data indicated that pre-participation levels of physical fitness of the participants of a SEP, when normalized for age and sex based on reference data, tend to be lower than these values. This is in accordance with the perspective that CVDs and other chronic degenerative diseases tend to be more prevalent in sedentary or low active individuals or in those with low physical fitness.

Our most important finding was that low, isolated, pre-participation levels of aerobic fitness, global FLX and body weight-related MPW seem to not influence medium-term adherence to a SEP. Even using a combined analysis of the extreme tertiles, we did not find any marked influence of the pre-participation levels of physical fitness components on medium-term adherence to the SEP, except for a practically irrelevant, borderline statistically significant difference between median P-MPW of 9 and 4 for the AD and NAD groups, respectively. In this context, it is worth pointing out that recent data have shown beneficial effects of a four-week CR program even on individuals aged older than 75 years, with coronary or valve disease, with improvement of aerobic fitness and MPW.^42^

The present study has a number of positive features that deserve considerations. First, there is a new appreciation of CR and its application in non-hospital approaches, including community programs like the SEP of this study.^43^ Second, our sample size of 567 participants was homogeneous regarding clinical features and regular use of medications after application of strict inclusion and exclusion criteria. Also, all measures were performed by only four physicians with wide experience in the protocols and measurement techniques, using routine assessment methods, which had been standardized in our lab. And since this was a retrospective study, the authors had no influence on the assessment and/or adherence to SEP results.

On the other hand, the study also has limitations that need to be addressed. Our sample included not only patients with coronary artery disease but also patients with many risk factors for CVD and other diseases. Unfortunately, we could not investigate objective indicators of the clinical reasons for SEP dropouts or mortality among participants of this study, which would be quite pertinent to the present study, and should be investigated in future studies. It is possible that the analysis of only some aspects of physical fitness lead to a limited and maybe biased view of the phenomenon of adherence to a SEP. However, the analysis of the extreme tertiles may corroborate our impression that pre-participation levels of the three physical fitness components, isolated or combined, do not affect medium-term adherence. Other aspects directly related to physical fitness, such as history of physical exercise and sports in different moments of life and magnitude of fitness gains during the SEP may have influenced adherence and should be object of future studies. In addition, racial, socioeconomic characteristics (most patients paid for participation in the SEP), and the higher proportion of men may have biased the present results and compromised their external validity. We could not assess the causes of SEP dropouts and whether participants who had dropped out the program before completing six months of participation continued or not to exercise by themselves and in different places such as clubs, gyms and even other SEPs. Further studies are needed to identify the influence of the components evaluated in this study, by comparing different programs and epidemiological profiles.

## Conclusion

The levels of pre-participation aerobic and non-aerobic physical fitness do not affect medium-term adherence to a SEP, although the knowledge of these levels is not only important but recommended for an individualized prescription of aerobic and non-aerobic exercises. This information reinforces the idea that patients with optimal physical fitness, and even debilitated patients or with low physical fitness can be referred for enrollment in a SEP by their assistant physicians and be adherent to the program for at least six months.
